# Hyaluronidases Have Strong Hydrolytic Activity toward Chondroitin 4-Sulfate Comparable to that for Hyaluronan

**DOI:** 10.3390/biom2040549

**Published:** 2012-11-12

**Authors:** Tomoko Honda, Tomoyuki Kaneiwa, Shuji Mizumoto, Kazuyuki Sugahara, Shuhei Yamada

**Affiliations:** 1 Laboratory of Proteoglycan Signaling and Therapeutics, Hokkaido University Graduate School of Life Science, Sapporo 001-0021, Japan; Email: ghk-gumi@ec.hokudai.ac.jp (T.H.); s22020109v@mail.sci.hokudai.ac.jp (T.K.); mizumoto@sci.hokudai.ac.jp (S.M.); sugar@sci.hokudai.ac.jp (K.S.); 2 Department of Pathobiochemistry, Faculty of Pharmacy, Meijo University, Nagoya 468-8503, Japan

**Keywords:** chondroitin sulfate, glycosaminoglycan, hyaluronan, hyaluronidase, hydrolase

## Abstract

Chondroitin sulfate (CS) chains are involved in the regulation of various biological processes. However, the mechanism underlying the catabolism of CS is not well understood. Hyaluronan (HA)-degrading enzymes, the hyaluronidases, are assumed to act at the initial stage of the degradation process, because HA is similar in structure to nonsulfated CS, chondroitin (Chn). Although human hyaluronidase-1 (HYAL1) and testicular hyaluronidase (SPAM1) can degrade not only HA but also CS, they are assumed to digest CS to only a limited extent. In this study, the hydrolytic activities of HYAL1 and SPAM1 toward CS-A, CS-C, Chn, and HA were compared. HYAL1 depolymerized CS-A and HA to a similar extent. SPAM1 degraded CS-A, Chn, and HA to a similar extent. CS is widely distributed from very primitive organisms to humans, whereas HA has been reported to be present only in vertebrates with the single exception of a mollusk. Therefore, a genuine substrate of hyaluronidases appears to be CS as well as HA.

## 1. Introduction

Chondroitin sulfate (CS) chains are linear polymers composed of the repeating disaccharide unit—4GlcUAβ1-3GalNAcβ1—where GlcUA and GalNAc represent D-glucuronic acid and *N*-acetyl-D-galactosamine, respectively, which are sulfated at different positions in various combinations [1,2]. CS chains have been demonstrated to be involved in the regulation of various biological processes such as cell proliferation, differentiation, and migration, cell-cell recognition, extracellular matrix deposition, and tissue morphogenesis [2,3,4]. The biological functions involved in such events have attracted much attention to the mechanism of CS biosynthesis [5]. However, not only biosynthesis but also catabolism is important for the regulation of the biological functions of CS.

**Figure 1:**
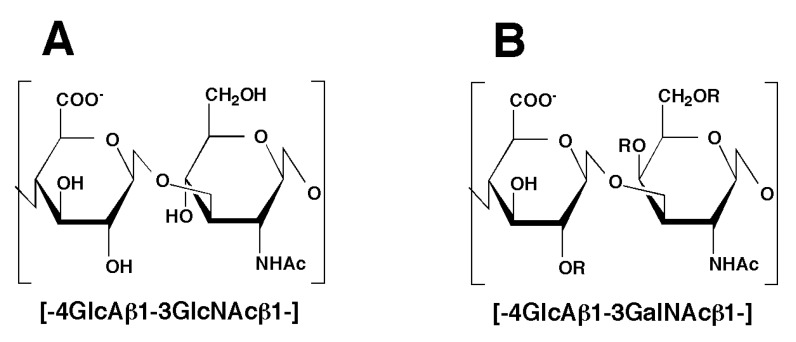
Typical structure of the disaccharide units found in hyaluronan (HA) and chondroitin sulfate (CS) chains. The backbone of HA (A) and CS (B) chains is a linear polymer composed of the repeating disaccharide unit, -4GlcAβ1-3GlcNAcβ1- and -4GlcAβ1-3GalNAcβ1-, respectively. Sugar residues of CS can be modified by ester sulfate at the C-2 position of GlcA and/or C-4 and/or C-6 of GalNAc as indicated by “R”.

The cellular degradation of CS occurs predominantly in lysosomes [6]. Following the fragmentation of polysaccharides by an endo-type hydrolase, the oligosaccharide products are degraded sequentially from the nonreducing end by exo-type glycosidases and sulfatases to liberate monosaccharide moieties in lysosomes. Hyaluronan (HA)-degrading enzymes, hyaluronidases, are considered to act at the initial stage of the degradation process, because HA is similar in structure to nonsulfated CS, chondroitin (Chn) (Figure 1). Since some hyaluronidases can degrade not only HA but also CS [7,8,9], the cellular degradation of CS is considered to be executed by hyaluronidases. 

Recently, we have identified human hyaluronidase-4 (HYAL4) as a CS-specific endo-β-*N*-acetylgalactosaminidase [10,11]. HYAL4 exhibited hydrolytic activity toward CS chains and degraded them into oligosaccharides, but hardly degraded HA at all. However, the expression of HYAL4 mRNA is not ubiquitous but restricted to placenta, skeletal muscle, and testis [12,13], suggesting that HYAL4 is not the enzyme involved in the systemic catabolism of CS, but rather has specific functions in particular organs or tissues. The biological functions of HYAL4 are yet to be clarified. 

Although hyaluronidase-1 (HYAL1) and testicular hyaluronidase (SPAM1) have been reported to degrade CS chains to some extent [7,8,9,14,15], their activity toward CS has not been determined precisely. The preferred substrate of HYAL1 and SPAM1 is considered to be HA rather than CS, but the kinetic parameters of these enzymes toward HA and CS have not been compared. In addition, the structural heterogeneity of the sulfation of CS makes it intrinsically more difficult to determine the hydrolytic activity toward CS. Although HA is composed of a simple, nonsulfated disaccharide unit—4GlcUAβ1-3GlcNAcβ1—the repeating disaccharide unit of CS is variously sulfated at different positions. The hydrolytic activity of a hyaluronidase toward CS-A, a major CS variant rich in GlcUAβ1-3GalNAc(4-*O*-sulfate) disaccharide units, might be distinct from that toward CS-C, another major variant rich in GlcUAβ1-3GalNAc(6-*O*-sulfate) disaccharide units. The degrading activities of hyaluronidases among these CS variants have not been quantitatively compared.

Hyaluronidase activity has been measured by HA substrate-based zymography [16], a turbidity assay [17,18], an ELISA-like assay using a HA-binding protein [19], agarose-gel electrophoresis with a fluorophore-labeled HA [20], and colorimetric methods using *p*-dimethylaminobenzaldehyde [21] and Alcian blue [22]. However, the HA zymography and ELISA-like assay are specific methods for the detection of HA-degrading activity and not suitable for measurements of CS-degrading activity. Since the turbidity assay and zymography are applicable only to high-molecular-weight substrates such as HA, they are not useful for the relatively small CS chains. Therefore, most of these assays are not quantitative for the measurement of CS-degrading activity. The measurement of reducing ends of resulting sugar oligomers by labeling with *p*-dimethylaminobenzaldehyde is quantitative but less sensitive. Thus, this colorimetric method is not suitable for a microanalysis of CS-degrading activity.

During the identification of HYAL4 as a CS-specific hydrolase [10,11], a new method has been developed to determine glycosaminoglycan-hydrolyzing activity with high sensitivity. Using this quantitative assay, we have in the present study measured and compared the hydrolytic activities of HYAL1 and SPAM1 toward CS variants as well as HA. Both HYAL1 and SPAM1 turned out to act on CS-A as well as on HA to a similar extent.

## 2. Results

### 2.1. Hydrolytic Activity of HYAL1 and SPAM1 toward CS Variants as well as HA

**Figure 2:**
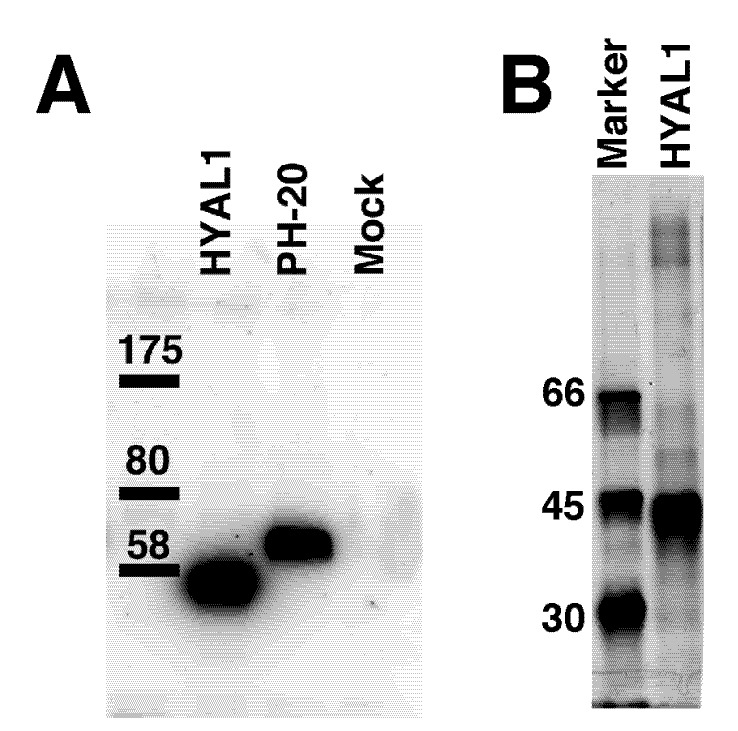
Expression of hyaluronidase-1 (HYAL1) and testicular hyaluronidase (SPAM1) in COS-7 cells. The culture medium of COS-7 cells was transfected with *HYAL1* or *SPAM1*, or mock-transfected cells was purified with ANTI-FLAG M2 affinity gel. The resin was subjected to sodium dodecyl sulfate polyacrylamide gel electrophoresis (SDS-PAGE) under reducing conditions and analyzed by Western blotting with the anti-FLAG antibody (A). The apparent molecular masses of the protein standards (right) are indicated. No bands were observed in the mock-transfected samples (left), suggesting that the bands detected in the lanes of HYAL1 and SPAM1 are their protein bands, respectively. The recombinant HYAL1 protein was eluted from the resin and subjected to SDS-PAGE. The purity wasexamined by silver staining (B). Molecular size marker proteins are shown on the left.

The hydrolytic activity of HYAL1 and SPAM1 for HA and the CS variants was compared. The enzymes were expressed in COS-7 cells at 37 °C as a recombinant protein fused with the FLAG tag at the N-terminus. The fusion protein secreted into the medium was adsorbed onto an anti-FLAG affinity gel for eliminating endogenous glycosidases. The recombinant HYAL1 protein was eluted from the resin and used as an enzyme source. In contrast, the protein-bound resin was used for the measurement of the activity of SPAM1, because SPAM1 has been reported to be present on the plasma membrane of sperm not as a soluble enzyme [23]. The purity of the enzyme was examined by western blotting (Figure 2). In contrast to the sample from mock-transfected cells, the bands of HYAL1 and SPAM1 were detected at molecular masses of 45 and 55 kDa, respectively.

The recombinant proteins were assayed for the hydrolytic activity of HYAL1 or SPAM1 at 37 °C for 15 min and 60 min, respectively, using CS variants and HA with an average molecular mass of 35 kDa as substrates. The same amount of each substrate was used under the same conditions. Each digest was then derivatized with 2-aminobenzamide (2AB) and analyzed by anion-exchange HPLC after digestion with chondroitinase AC-II. Reactions with HYAL1 or SPAM1 were terminated while less than 5% of the substrate was consumed, suggesting that the substrate is always in excess. Since the increase in the amount of 2AB-labeled oligosaccharides after enzymatic digestion corresponds to that of the newly formed reducing ends, the fluorescent intensity of 2AB can be used to quantify the number of sites cleaved by the enzyme in HA and CS preparations.

**Figure 3:**
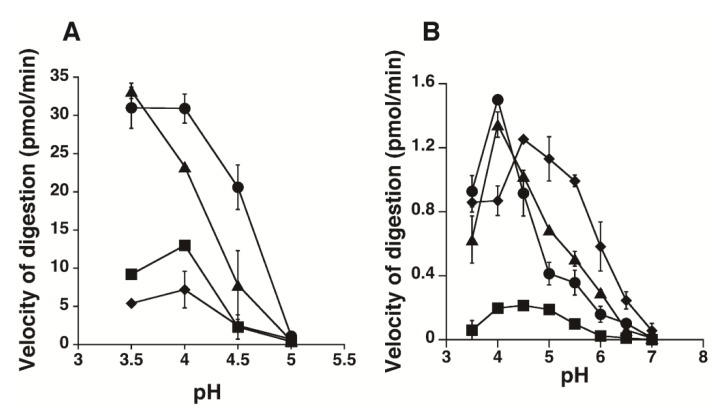
The pH profiles for the hydrolytic activity of HYAL1 (A) and SPAM1 (B). CS-A (circles), CS-C (squares), chondroitin (Chn) (diamonds), or HA (triangles) wereincubated with purified HYAL1 or SPAM1 in 50 mM formate buffer, pH 3.5–5.0, or 50 mM formate buffer, pH 3.5–5.0, and 50 mM phosphate buffer, pH 5.0–7.0, respectively, containing 150 mM NaCl and the digests were labeled with 2-aminobenzamide (2AB). The 2AB-derivatives were digested with chondroitinase AC-II, and then analyzed by anion-exchange high performance liquid chromatography (HPLC) on an amine-bond silica column. Degradation of CS variants and HA was assessed by velocity of digestion as described under the “Experimental Section”. The experiments were performed under conditions in which less than 5% of total substrate was digested. Values represent the mean ± SD (n = 3).

The enzymatic activity of HYAL1 or SPAM1 towards Chn, CS-A, CS-C, and HA (35 kDa) was examined under various pH conditions (from 3.5 to 7.0), and the initial velocity of the digestion was determined (Figure 3). Unexpectedly, HYAL1 hydrolyzed CS-A at a higher velocity than HA at pH 4.0–4.5. The relative rate of degradation of HA (35 kDa) : CS-A : CS-C : Chn by HYAL1 at pH 4.0 was 1.0 : 1.3 : 0.6 : 0.3, respectively. HYAL1 showed weak but significant activity toward CS-C, but hardly acted on Chn (Figure 3A). The optimum pH for the activity of HYAL1 toward the CS variants was different from that toward HA. Although HYAL1 exhibited the highest hydrolytic activity toward the CS variants at pH 4.0, the rate of degradation of HA by HYAL1 still increased below pH 4.0 (Figure 3A). pH-dependent conformational alterations in the HYAL1 protein may have contributed to the recognition of sulfate groups in CS.

Compared with HYAL1, SPAM1 was active throughout a wide pH range. SPAM1 also exhibited higher hydrolytic activity toward CS-A than HA (35 kDa) at pH 4 (Figure 3B). The relative rate of degradation of HA (35 kDa) : CS-A : CS-C : Chn by SPAM1 at pH 4.0 was 1.0 : 1.1 : 0.1 : 0.6, respectively. In contrast to HYAL1, SPAM1 hardly depolymerized the CS-C variant. The pH-dependent degradation of Chn by SPAM1 was unique. Compared with the optimum pH of SPAM1 for HA and CS-A (pH 4.0), that for Chn was in the higher range (pH 4.5–5.5). The relative rate of degradation of HA (35 kDa) : CS-A : CS-C : Chn by SPAM1 at pH 5.5 was 1.0 : 0.7 : 0.2 : 2.0, respectively. SPAM1 shows a distinct substrate preference depending on pH. It prefers HA and CS to Chn at pH 4.0, whereas this preference becomes reversed above pH 4.5. 

**Table 1:** Kinetic parameters of the recombinant HYAL1.

Substrate	Apparent *K*_m_	Apparent *V*_max_	Apparent *V*_max_*/K*_m_
	mM as disaccharides	pmol/min	
HA	0.40	23.0	57.5
CS-A	0.21	30.5	145
CS-C	0.64	10.8	16.9
Chn	1.20	9.0	7.5

**Table 2:** Kinetic parameters of the recombinant SPAM1.

Substrate	Apparent *K*_m_	Apparent *V*_max_	Apparent *V*_max_*/K*_m_
	mM as disaccharides	pmol/min	
HA	0.20	0.92	4.5
CS-A	0.13	0.94	7.5
CS-C	0.48	0.28	0.6
Chn	1.02	1.55	1.5

**Figure 4:**
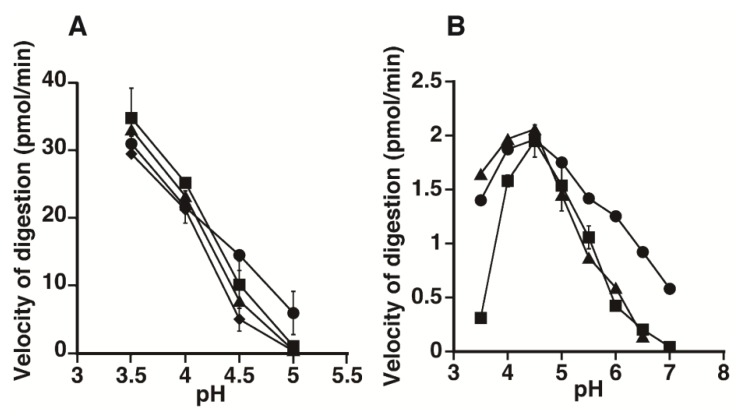
The pH profiles for the hydrolytic activity of HYAL1 (A) and SPAM1 (B) towards HA preparations with different molecular masses. The HA preparations, which had an average molecular mass of 6.5 (circles), 130 (squares), or 1,000 (diamonds) kDa, were incubated with purified HYAL1 or SPAM1 in 50 mM formate buffer, pH 3.5–5.0, or 50 mM formate buffer, pH 3.5–5.0, and 50 mM phosphate buffer, pH 5.0–7.0, respectively, containing 150 mM NaCl and the digests were labeled with 2AB. The 2AB-derivatives were digested with chondroitinase AC-II, and then analyzed by anion-exchange HPLC on an amine-bond silica column. Degradation was assessed by velocity of digestion. The experiments were performed under conditions in which less than 5% of total substrate was digested. Values represent the mean ± SD (n = 3).

To examine the effects of the chain length of HA on the susceptibility to the enzymes, the HA preparations, which have an average molecular mass of 6.5 or 130 kDa from *Streptococcus pyogenes* or 1,000 kDa from human umbilical cord, were also incubated with HYAL1 or SPAM1 under various pH conditions (Figure 4). However, no significant difference was observed in the amount of oligosaccharides formed, suggesting that the chain length of HA does not strongly affect the specificity of either recombinant enzyme.

### 2.2. Kinetic Analysis of CS-Degrading Activity of HYAL1 and SPAM1

**Figure 5:**
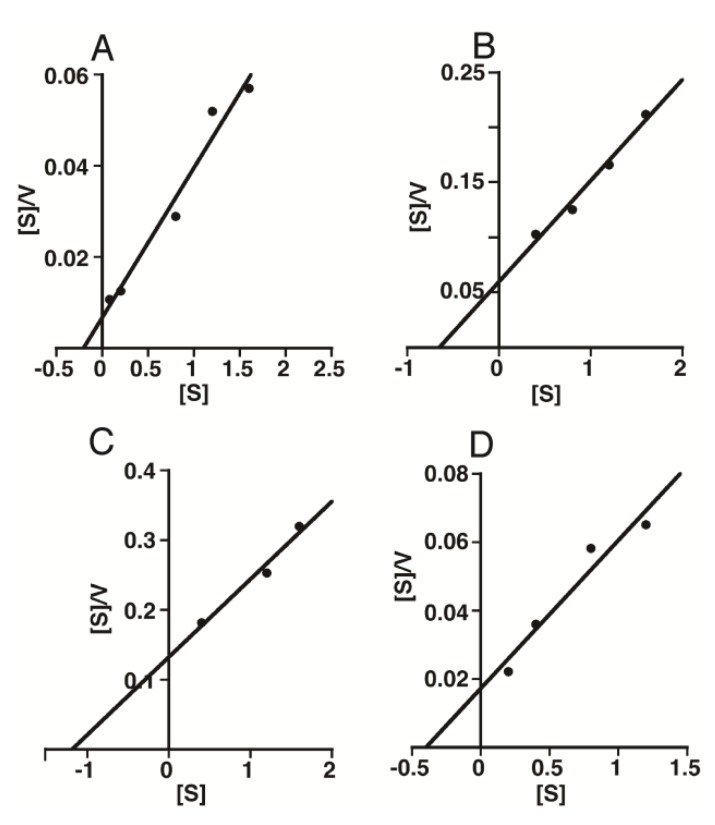
Hanes-Woolf plots of the initial velocities obtained by enzymatic hydrolysis of varying concentrations of CS isoforms and HA with HYAL1. HYAL1 was incubated in 50 mM formate buffer, pH 4.0, with different concentrations of CS-A (A), CS-C (B), Chn (C), or HA (D). After incubation, degradation products were labeled with 2AB and digested with chondroitinase AC-II. The resultant oligosaccharides were quantified by HPLC as described in the “Experimental Section”. The plots show linearity and the reaction velocity was used for a kinetic analysis to determine apparent *K_m_*, *V_max_*, and *V_max_*/*K_m_* values for HYAL1.

**Figure 6:**
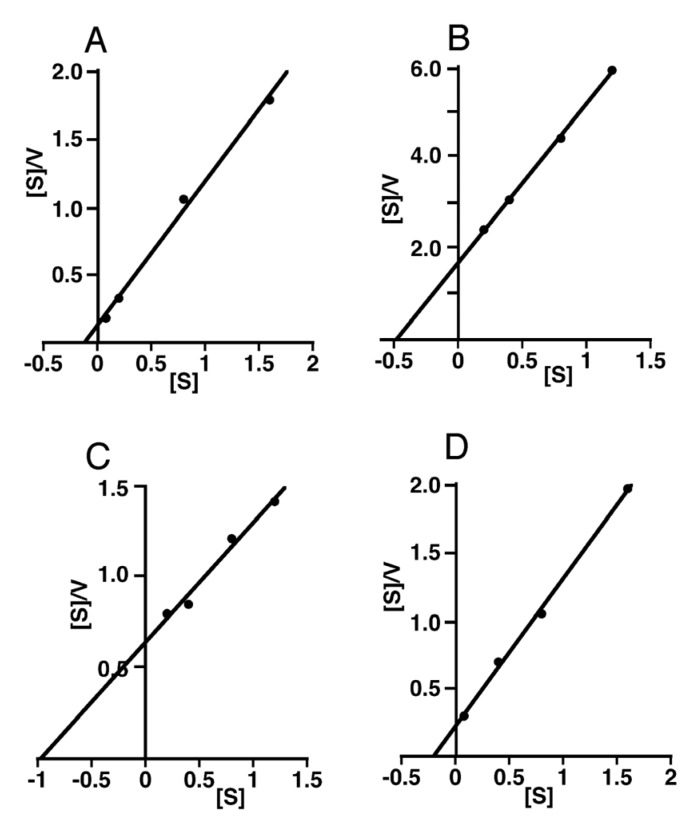
Hanes-Woolf plots of the initial velocities obtained by enzymatic hydrolysis of varying concentrations of CS isoforms and HA with SPAM1. SPAM1 was incubated in 50 mM formate buffer, pH 4.0, with different concentrations of CS-A (A), CS-C (B), Chn (C), or HA (D). For details, see the legend to Figure 5.

To compare the enzymatic activity of HYAL1 or SPAM1 towards different substrates in more detail, a kinetic analysis was performed. As both enzymes exhibited strong activity towards HA and CS around pH 4.0, experiments were conducted at pH 4.0. The initial reaction rates and substrate concentrations (as disaccharide) were used for an analysis with Hanes-Woolf plots (Figure 5 and Figure 6). The apparent Michaelis-Menten constants as well as *V*_max_ values for Chn, CS-A, CS-C, and HA with an average molecular mass of 35 kDa were determined and are shown in Table 1 and Table 2. The apparent *K*_m_ values of HYAL1 were similar among the CS variants and HA (35 kDa), indicating that HYAL1 equally recognizes them as its substrates. However, the apparent *V*_max_ values of HYAL1 toward HA (35 kDa) and CS-A were higher than those toward CS-C and Chn (Table 1). This may contribute to the difference in the degradation rate of HYAL1 among these substrates. In fact, the substrates ranked as follows in order of the ratio *V*_max_/*K*_m_: CS-A>HA>CS-C>Chn, being consistent with the order of the initial reaction rates at pH 4.0 calculated in Figure 3A.

The apparent *K*_m_ values of SPAM1 for HA (35 kDa) and CS-A were several times smaller than those for CS-C and Chn (Table 2), suggesting that SPAM1 has a higher affinity for the former substrates than the latter. In addition, the apparent *V*_max_ values of SPAM1 for HA (35 kDa), CS-A, and Chn were several times higher than those for CS-C (Table 2). When the apparent *V*_max_/*K*_m_ values of SPAM1 were compared, the substrates ranked as follows: CS-A>HA>Chn>CS-C. This order was the same as that for the initial reaction rates of SPAM1 at pH 4.0 calculated in Figure 3B.

## 3. Discussion

The genuine substrate of hyaluronidases has been considered to be HA, although the enzymes also depolymerize CS, because HYAL1 was reported to digest CS more slowly than HA [8,9]. Therefore, we have sought a CS-specific endo-type hydrolase [24,25], and demonstrated HYAL4 to be a CS-specific hydrolase [10,11]. However, the expression of human HYAL4 is not ubiquitous but restricted to the placenta, skeletal muscle, and testis [12,13]. HYAL4 does not seem to be involved in the systemic catabolism of CS in lysosomes, but rather has specific temporal functions in particular organs or tissues. How are CS chains systemically degraded under physiological conditions? Since HYAL1 is ubiquitously expressed and can degrade CS chains to only a limited extent [7,8,9], we estimated that it might depolymerize CS chains under specific pH conditions or by forming a complex with some other assistant protein(s). In fact, the HA-degrading activity of HYAL2 at pH 6.0-7.0 was detected only when the enzyme was co-expressed with CD44 [26]. In addition, no quantitative comparison of the hydrolytic activity of hyaluronidases toward CS and HA had ever been reported to our knowledge. 

In the present study, the hydrolytic activities of HYAL1 and SPAM1 toward CS-A, CS-C, Chn, and HA have been determined. Unexpectedly, both HYAL1 and SPAM1 depolymerized CS-A as well as HA at a similar velocity at pH 4.0 (Figure 3). Under conditions of pH 4.5, HYAL1 preferentially recognizes CS-A rather than HA, although it degrades HA at a higher velocity than CS-A at pH 3.5 (Figure 3A). In regard to degradation by HYAL1 and SPAM1, CS-A turned out to be comparable to HA. Furthermore, the optimal pH of SPAM1 was higher for Chn than HA and CS-A. SPAM1 prefers Chn to HA and CS-A under higher pH conditions (Figure 3B). In contrast, the digestion of CS-C by HYAL1 and SPAM1 was much slower than that for other substrates, indicating that the 6-*O*-sulfate group on the GalNAc residue has inhibitory effects on hyaluronidases. It should be noted, however, that the enzymes used in this study were not the native protein but fused with the FLAG tag. The possibilities cannot be excluded that the presence of the octapeptide DYKDDDDK sequence may influence the activity of the enzymes and that the activities toward the different substrates as well as the pH optima may be altered from those of the native enzymes.

Since HYAL1 is ubiquitously expressed and hydrolyzes CS-A and HA to a similar extent, it is a candidate for the enzyme involved in the systemic catabolism of CS, as the earlier investigators estimated. The major disaccharide unit found in mammalian CS is the GalNAc(4-*O*-sulfate)-containing disaccharide unit (CS-A type) [27,28], being consistent with the substrate preference of HYAL1. It, however, should be noted that a significant amount of the GalNAc(6-*O*-sulfate)-containing disaccharide unit (CS-C type) is also present in mammals. CS-C isoforms might be hydrolyzed by other HYAL family members or the substrate-specificity of HYAL1 might be modified to recognize CS-C by forming a complex with some other assistant protein(s), as for the HA-degrading activity of a HYAL2/CD44 complex at pH 6.0–7.0 [26]. Another possibility is that exo-type CS-degrading enzymes may be able to sufficiently catabolize CS polysaccharide chains. Although the loss of exoglycosidases to hydrolyze glycosaminoglycan oligosaccharides leads to severe genetic disorders, mucoplysaccharidoses [6], a patient with a deficiency of HYAL1 was reported to show a mild clinical phenotype [29], suggesting that exo-type CS/HA-degrading enzymes play the major role, and the contribution by endo-glycosidases might be small in the catabolism of CS/HA.

When the apparent *V*_max_/*K*_m_ values of HYAL1 or SPAM1 for HA, CS-A, and Chn at pH 4.0 were compared, the substrates ranked as follows: CS-A>HA>Chn. Since the structure of the sugar backbone is the same between Chn and CS-A, the order was expected to be HA>Chn>CS-A or CS-A>Chn>HA. However, Chn was not in the middle position though it was less sensitive to HYAL1 than HA which is also nonsulfated but differs from Chn/CS-A in the structure of the sugar backbone. The pH profiles of the hydrolytic activity of SPAM1 toward HA and CS-A were similar but different from that toward Chn. Therefore, the steric conformation of HA and CS-A may resemble but be distinct from that of Chn.

Chn/CS most likely occurred in evolution prior to HA [30]. Although Chn and CS have been demonstrated in nematodes (Nematoda) [31] and hydrozoan (Cnidaria) [32], respectively, HA has not. HA appears to be common among metazoan vertebrates [30]. Some bacterial strains can also synthesize HA when they form extracellular polysaccharide capsules [33]. However, HA appears to be an animal innovation. Isolation of HA has also been reported in mollusks, but only in the bivalve *Mytilus galloprovincialis* [34]. Therefore, HA most likely emerged at a relatively late stage of evolution. Previously we demonstrated the human hyaluronidase homolog in *Caenorhabditis elegans* to be a Chn hydrolase [24]. Based on the appearance of CS/Chn prior to HA during evolution, hyaluronidases seem to be originally Chn/CS hydrolases and to have acquired the hydrolytic activity toward HA later. Thus, the genuine substrate of “hyaluronidases” used to be CS during the early stages of evolution, and that is why HYAL1 and PH20 appear to be able to act on not only HA but also CS-A to a similar extent.

The sugar stereoconfiguration, the substitution pattern of the backbone hydroxy groups, and the glycosidic linkages are identical in Chn and HA. The only difference in structure is the configuration at the C-4 position of the hexosamine residues. Although HYAL1 degrades not only HA but also CS/Chn, HYAL4 is specific for CS/Chn [10,11]. The amino acid residues responsible for distinguishing GalNAc from GlcNAc have yet to be identified. Studies of sequence homology between HYAL4 and HYAL1 may provide some information concerning those amino acids. Based on the three-dimensional modeling of human HYALs, Jedrzejas and Stern [9] have claimed that Cys^263^ in HYAL4 replaced by Tyr^247^ in HYAL1 may cause the difference in substrate specificity. The Cys^263^ in HYAL4 reported by Jedrzejas and Stern [9] (accession number: AF009010) was replaced by Gly^263^ in the sequence of the HYAL4 gene cloned by us (accession number: AB470346) and the Mammalian Gene Collection Program Team (accession numbers: BC104788 and BC104790) as well as in human genomic DNA (accession number: NT_007933). It, however, should be noted that the Tyr^247^ residue in HYAL1 is conserved among all HYAL family members except for HYAL4. In the sequence of the CS-specific mouse Hyal4 gene, the corresponding Tyr residue is replaced by Ser^263^. To investigate the involvement of Tyr in the recognition of GlcNAc, point mutants, human HYAL4(Gly^263^to Tyr) as well as mouse Hyal4(Ser^263^ to Tyr), were generated and their substrate specificity was characterized (Kaneiwa, Sugahara, and Yamada*,* unpublished). Based on the unpublished observations, mouse Hyal4(Ser^263^ to Tyr) hydrolyzed not only CS but also HA, indicating that replacement of Ser by Tyr conferred the HA-degrading activity to mHyal4. However, human HYAL4(Gly^263^to Tyr) still depolymerized only CS and not HA. The Tyr residue in HYAL1 appears to be partially involved in the recognition of GlcNAc in HA, but it is not sufficient to govern the substrate specificity. Identification of the amino acid residues responsible for the recognition of the amino sugars may lead to the development of artificial CS/HA hydrolases, which recognize specific CS structures or HA and will be useful for elucidating novel roles of CS or HA.

## 4. Experimental Section

### 4.1. Materials

The following sugars and enzymes were purchased from Seikagaku Corp. (Tokyo, Japan): CS-A from whale cartilage, CS-C from shark cartilage, Chn, a chemically desulfated derivative of CS-A, chondroitinase ABC from *Proteus vulgaris* (EC 4.2.2.20), and chondroitinase AC-II from *Arthrobacter aurescens* (EC 4.2.2.5). HA from human umbilical cord was obtained from Sigma (Saint Louis, MO, USA). The average molecular size of the commercial Chn, CS-A, CS-C, and HA from human umbilical cord was determined to be 23, 34, 64, and 1,000 kDa, respectively, by gel filtration chromatography with size-defined dextran preparations as standards (data not shown). HA preparations, which have an average molecular mass of 6.5, 35, or 130 kDa, from *Streptococcus pyogenes* were purchased from R&D systems, Inc. (Minneapolis, MN, USA). COS-7 cells were obtained from Japan Health Sciences Foundation (Tokyo, Japan). The pCMV-SPORT6/human HYAL1 vector (IMAGE Consortium cDNA clone, ID number 5186626) was from ResGen (Huntsville, AL, USA). Human testis cDNA was purchased from Clontech.

### 4.2. Cloning of SPAM1 cDNA

The putative full-length open reading frame encoding human SPAM1 was amplified from the human testis cDNA by two rounds of PCR using specific primers corresponding to the sequences in the 5’- and 3’-noncoding regions. The first PCR was performed with the primers, 5’-GGT CCT TCC TAG CAA GGG ATG CTA A-3’ and 5’-GCA CTT AAT ACC CAA GGA TGT TGG-3’. The second PCR was performed with the nested primers, 5’-GAG ACC AGC CAA CTT CTT GCC TTG-3’ and 5’-TGT AAG CCA AGG GAA GAG GCC TG-3’. Each PCR was carried out with the KOD-Plus DNA polymerase (Toyobo, Tokyo, Japan) in the presence of 5% (v/v) dimethyl sulfoxide for 30 cycles at 95 °C for 30 s, 54 °C or 57 °C for 42 s, respectively, and 68 °C for 2 min. The amplified cDNA fragment of expected size (~2.0 kbp) was subcloned into a pGEM®-T Easy vector (Promega) and sequenced by Hokkaido System Science (Sapporo, Japan).

### 4.3. Construction of an Expression Vector Containing a cDNA Fragment Encoding a Soluble Form of HYAL1 or SPAM1

The DNA fragment which encodes the HYAL1 protein lacking the first *N*-terminal 28 amino acids (a hydrophobic region), was amplified by PCR with the pCMV-SPORT6/human HYAL1 vector as a template, using a 5’ primer containing an in-frame *Eco*RV site (5’-GCG ATA TCG AAC CGG CCC TTC ACC ACC-3’) and a 3’ primer containing a *Bam*HI site (5’-GCG GAT CCG CAT TAG GTT CTC AAT AT-3’). PCR was carried out with the KOD-Plus DNA polymerase for 30 cycles at 95 °C for 30 s, 55 °C for 45 s, and 68 °C for 2 min. The DNA fragment which encodes the SPAM1 protein lacking both the first *N*-terminal 36 amino acids (a hydrophobic region), and the last C-terminal 26 amino acids (the putative glycosylphosphatidylinositol-anchored region) was amplified by PCR with the pGEM^®^-T Easy vector containing the *SPAM1* gene as a template, using a 5’ primer containing an in-frame *Eco*RV site (5’-GCG ATA TCG AAT TTC AGA GCA CCT CCT-3’) and a 3’ primer containing a *Bam*HI site (5’-GCG GAT CCT CAA GCA TTG TAG AAA ATT TG-3’). PCR was carried out with the KOD-Plus DNA polymerase for 30 cycles at 95 °C for 30 s, 56 °C for 45 s, and 68 °C for 2.5 min. The amplified fragment was cloned into the *Eco*RV and *Bam*HI sites of the expression vector p3XFLAG-CMV-8 (Sigma), resulting in the fusion of HYAL1 or SPAM1 to the preprotrypsin leader sequence and the FLAG tag sequence present in the vector. The preprotrypsin leader sequence was used for the effective expression of the enzyme protein instead of the original signal sequence.

### 4.4. Expression of a Soluble Form of HYAL1 or SPAM1

The expression plasmids (6.7 μg) were introduced into COS-7 cells using FuGENE^TM^6 (Roche Diagnostics, Basel, Switzerland) according to the manufacturer’s instructions. After 3 days of culture at 37 °C, 8 mL of the culture medium was collected and incubated with 160 μL of anti-DYKDDDK antibody-conjugated affinity gel (Wako) overnight at 4 ˚C. The resin was washed with 25 mM Tris-buffered saline containing 0.05% Tween-20. The recombinant HYAL1 protein was eluted from the resin with 400 μL of 0.1 M glycine-HCl buffer (pH 2.7) and the eluate was neutralized with Tris-HCl buffer (pH 9.5).

### 4.5. Measurement of the Enzymatic Activity

The SPAM1-bound resin was washed with an incubation buffer and then resuspended with 64 μL of the buffer. The suspension (14 μL) was incubated with various concentrations of CS variants or HA at 37 °C in a 50 mM formate or phosphate buffer, pH 3.0–7.0, containing 150 mM NaCl in a total volume of 40 μL, and the reactions were terminated at 60 min. The purified HYAL1 protein (5 μL as the eluate) was also incubated with various substrates, and the reactions were terminated at 15 min. The reaction mixture was occasionally mixed with a vortex mixer. The SPAM1-bound resin was removed by filtration using an Ultrafree-MC filter (Millipore, Billerica, MA, USA). Each sample was labeled with 2AB [35], and excess 2AB-derivatizing reagents were removed by extraction with chloroform [36]. The 2AB-derivatives were digested by chondroitinase AC-II (5 mIU) in 50 mM sodium acetate buffer, pH 6.0, and the digests were analyzed by anion-exchange HPLC on an amine-bound silica PA-03 column (4.6 × 250 mm, YMC Co., Kyoto, Japan) using a linear gradient of NaH_2_PO_4_ from 16 to 538 mM over 60 min at a flow rate of 1 mL/min. Eluates were monitored by measuring fluorescence with excitation and emission wavelengths of 330 and 420 nm, respectively. The hydrolytic activity of HYAL1 or SPAM1 was assessed based on the velocity of digestion, which was determined by measuring the peak area. The reaction rate was measured as moles of the products formed/min and apparent Michaelis-Menten constants were determined by fitting the data to the Hanes-Woolf equation ([S]/V = [S]/*V_max_* + *K_m_*/*V_max_*).

### 4.6. Western Blotting

The enzyme-bound resins were washed with 25 mM Tris-buffered saline containing 0.05% Tween-20, and incubated with the SDS sample buffer and DTT solution (New England Biolabs) in a total volume of 30 μL at 100 °C for 5 min. After centrifugation at 1,000 rpm for 2 min, the supernatant fluid was subjected to SDS-PAGE. Proteins were resolved on 7.5% SDS-polyacrylamide gels and transferred to a polyvinylidene difluoride membrane. The membrane was incubated with peroxidase-labeled anti-DYKDDDDK monoclonal antibody (Wako) diluted 1:1,000 with the blocking buffer overnight. The bound antibody was detected using an Immunostar LD (Wako). The purity of the enzyme was also examined by silver staining as described [24].

## 5. Conclusions

In this study we have determined the hydrolytic activity of HYAL1 as well as the testicular hyaluronidase, SPAM1, quantitatively. Their activities toward CS-A were demonstrated to be comparable to those toward HA. This is the first characterization of the activity of these hyaluronidases toward CS. CS chains may be the primary substrate for both hyaluronidases. Recently, Gushulak *et al.* [37] reported the accumulation of CS in mice deficient in Hyal1 and β-hexosaminidase, indicating the *in vivo* function of HYAL1 in the systemic degradation of CS. This was consistent with the present observation that HYAL1 has strong hydrolytic activity toward CS-A. HYAL1 appears to be involved in various biological processes by degrading not only HA but also CS chains. It is important to elucidate the function and influence of the degradation of CS by hyaluronidases *in vivo* under physiological as well as pathological conditions.

## Abbreviations

2AB: 2-aminobenzamide
Chn: chondroitin
CS: chondroitin sulfate
HA: hyaluronan
HPLC: high performance liquid chromatography
